# Digital Image Analysis for the Detection of Group B *Streptococcus* from ChromID Strepto B Medium Using PhenoMatrix Algorithms

**DOI:** 10.1128/JCM.01902-19

**Published:** 2020-12-17

**Authors:** Justin Baker, Karen Timm, Matthew Faron, Nathan Ledeboer, Karissa Culbreath

**Affiliations:** a TriCore Reference Laboratories, Albuquerque, New Mexico, USA; b Department of Pathology and Laboratory Medicine, Medical College of Wisconsin, Milwaukee, Wisconsin, USA; c Department of Pathology, University of New Mexico, Albuquerque, New Mexico, USA; Marquette University

**Keywords:** artificial intelligence, chromogenic media, group B streptococcus, total laboratory automation

## Abstract

Group B *Streptococcus* (GBS) can be found to colonize about 25% of all healthy, adult women and is the leading infectious cause of early neonatal morbidity and mortality in the United States. This study evaluated the clinical performance of PhenoMatrix (PM) chromogenic detection module (CDM) digital imaging software in detection of GBS from LIM broth plated on ChromID Strepto B chromogenic medium (ChromID) using the WASP automated processor. The performance of the PM CDM was compared to manual culture review of the digital images and molecular detection of GBS. ChromID alone had a sensitivity and specificity of 84.5% and 94.7%, respectively, after 48 h compared to nucleic acid amplification testing (NAAT). Compared to the composite reference for positivity, when PM CDM was used to detect GBS from ChromID, the sensitivity was 100%, with no true-positive GBS isolates missed by 48 h of incubation. Overall, evaluating all three methods for the detection of GBS, the sensitivities of NAAT, ChromID plus PM CDM at 48 h, and ChromID alone at 48 h were 96.8%, 95.5%, and 90.3%, respectively. The specificities of NAAT, ChromID plus PM CDM, and ChromID alone were 97.7%, 63.0%, and 95.4%, respectively. The sensitivity of ChromID in combination with the PM CDM was similar to the sensitivity of molecular detection. Further, the algorithm never called a culture negative that was determined to be positive by manual reading, and it identified an additional eight true positive specimens that were missed by manual digital image culture reading.

## Abstract (precis)

Group B *Streptococcus* (GBS) can be found to colonize about 25% of all healthy, adult women and is the leading infectious cause of early neonatal morbidity and mortality in the United States. This study evaluated the clinical performance of PhenoMatrix (PM) chromogenic detection module (CDM) digital imaging software in detection of GBS from LIM broth plated on ChromID Strepto B chromogenic medium (ChromID) using the WASP automated processor. The performance of the PM CDM was compared to manual culture review of the digital images and molecular detection of GBS.

## INTRODUCTION

Group B *Streptococcus* (GBS) has been recognized as a leading cause of infectious early neonatal morbidity and mortality in the United States and around the world (1). Patients typically present with respiratory distress, apnea, or other constitutional signs of sepsis, with mortality from early-onset GBS ranging from 2 to 20% and the highest rates being among infants born following less than 33 weeks of gestation (2). Approximately 10 to 30% of pregnant women are colonized with GBS during pregnancy, which can be transmitted to the infant at birth (3). Of note, the American College of Obstetricians and Gynecologists recently updated the previous CDC recommendations for universal screening from 35 to 37 weeks of gestation to 36 to 37 weeks of gestation (4). Following the first recommendations for screening, the rate of early-onset GBS disease has decreased from 1.8 cases per 1,000 live births to 0.23 case per 1,000 live births (2015) (4).

Culture of LIM broth on blood-containing medium is the gold standard for detection of GBS. Use of selective or chromogenic medium has increased the sensitivity of culture for GBS, but these methods may still be less sensitive than nucleic acid amplification tests (NAATs) for the detection of GBS (5–8). There are several commercially available NAATs for the detection of GBS that increase sensitivity, require less hands-on time, and provide a faster result, but they are often more expensive and may have slightly lower specificity than culture (9, 10).

Although the use of NAAT for detection of GBS is increasing, a recent CDC survey demonstrated that only 18.7% of laboratories reported using NAAT for GBS screening, which may be due to the increased costs associated with this technology (11). Thus, continued improvement of culture methods for diagnosis of GBS is imperative.

Copan’s PhenoMatrix (PM) software (Copan Diagnostics, Murrieta, CA) is a suite of advanced artificial intelligence algorithms with the ability to preassess and presort culture plates based on its “reading” of digital plate images. For example, the software can segregate urine culture plates according to whether they show growth, no growth, or insignificant growth, and the color detection module (CDM) can recognize and differentiate colony colors on chromogenic agars. Previous studies have described the ability of the PM CDM software to increase the sensitivity and specificity of detection of methicillin-resistant Staphylococcus aureus (MRSA), vancomycin-resistant *Enterococcus* (VRE), and group A *Streptococcus* using chromogenic media (12–14). PM software is designed to be an extension of the WASPLab. The WASPLab system is composed of an up-front specimen processing unit (walk-away specimen processor [WASP]) and an integrated track line(s) to automatically move inoculated culture plates from processing to incubation to bench. The system also includes a smart incubator(s) that provides constant atmospheric and temperature conditions for all plates, plus a digital imaging system to record images of plates at user-specified times. The technologists then view these digital images for culture work-up, rather than handling the plates themselves.

We evaluated the performance of the PM CDM software used with ChromID Strepto B medium for GBS (ChromID GBS; bioMérieux, Durham, NC) for the detection of GBS in LIM broths compared to routine visual inspection and a molecular detection method.

## MATERIALS AND METHODS

### Samples.

A total of 676 residual vaginal-rectal swabs in LIM broth (Remel, Lenexa, KS) were enrolled at two sites (TriCore Reference Laboratories, Albuquerque, NM, and Medical College of Wisconsin, Milwaukee, WI) in the study. LIM broths were incubated at 35 to 37°C for 18 to 24 h as per standard laboratory procedures. One milliliter of enriched LIM broth was aliquoted into empty sterile Copan 12- by 80-mm tubes for automated processing on the WASP (Copan Diagnostics, Murrieta, CA).

### GBS culture.

Portions (30 μl) of LIM broths were inoculated by the WASP onto one ChromID Strepto B chromogenic medium plate (bioMérieux) and a blood agar plate. Cultures were incubated in the WASPLab (Copan Diagnostics) incubator in ambient air at 35 to 37°C, with digital images captured at initial plating (0 h), 24 h, and 48 h.

### Culture reading.

Digital images of cultures were reviewed manually at 24 and 48 h by a technologist and scored for the presence or absence of colonies resembling GBS. As indicated by the manufacturer, morphologies consistent with GBS were confirmed using Gram stain, catalase reaction, and latex Lancefield grouping and/or matrix-assisted laser desorption ionization–time of flight (MALDI-TOF) identification according to standard operating procedures in the laboratory.

### PM CDM.

Digital images of cultures were also analyzed by PM CDM for the detection of GBS colonies. PM CDM was used to evaluate images of colonies for the appropriate coloration on the chromogenic agar indicating a possible GBS organism. It was then used to presort culture plates based on its reading of digital plate images. For example, the software recognized and differentiated colony colors on chromogenic agars and sorted the cultures into the categories “negative for GBS” and “potential positive for GBS.” Colonies detected as potentially positive by the PM CDM were also identified by conventional methods as described above.

### Molecular detection of GBS.

All enriched LIM broths were tested by BD MAX GBS (BD Diagnostics, Sparks, MD). Discordant results between the BD MAX GBS and the culture were resolved using a second NAAT, Cepheid Xpert GBS (Cepheid, Sunnyvale, CA).

### Data analysis.

A composite reference was used to determine true positivity and to adjudicate discordant results. A specimen was considered a true-positive GBS if a colony derived by any method was confirmed to be GBS by MALDI-TOF or if both molecular methods were positive for GBS. The result was considered a true negative if the presumptive positive colony was not confirmed as GBS by MALDI-TOF or if only one of the two molecular methods was positive. To evaluate the performance between the culture methods and NAAT, the sensitivity, specificity, positive predictive value (PPV), and negative predictive value (NPV) were determined by comparing the result of each method to the composite reference result.

## RESULTS

To determine the overall performance of the ChromID GBS agar, it was compared to the BD MAX GBS assay. The ChromID GBS demonstrated a sensitivity at 24 h of 72.1% (Table 1). Compared to NAAT alone, we observed a difference in specificity of ChromID GBS at 48 h (94.8%) and at 24 h (97.7%) (Table 1). However, at 48 h, 20 additional true-positive cultures were detected, for a sensitivity of 84.5% compared to NAAT alone (Table 1). Twenty-seven samples were negative by NAAT and positive by chromogenic agar culture at 48 h. Of these samples, 24 were false positive by the chromogenic medium. The organisms causing the false-positive chromogenic reactions were Streptococcus anginosus (3 samples), Streptococcus viridans (3 samples), Enterococcus faecium (5 samples), Enterococcus avium (1 sample), Streptococcus mitis*/*Streptococcus oralis (6 samples), and other *Streptococcus* species (2 samples); an additional 4 falsely positive chromogenic cultures were unavailable for organism identification. Three of the 27 chromogenic culture-positive samples that were missed by NAAT were considered true positives, resulting in 139/154 positive GBS samples detected by the chromogenic medium at 48 h of incubation.

**TABLE 1:** Performance of the ChromID GBS culture compared to GBS NAAT alone with culture reading at 24 and 48 h of incubation

Incubation time (h)	ChromID result	No. of samples with NAAT result		% (95% CI)			
		Positive	Negative	Sensitivity	Specificity	PPV	NPV
24	Positive	116	12	72.1 (64.3–78.7)	97.7 (95.9–98.7)	90.6 (83.9–94.8)	91.8 (89.1–93.9)
	Negative	45	503				
48	Positive	136	27	84.5 (77.8–89.5)	94.7 (92.4–96.5)	83.4 (76.6–88.6)	95.1 (92.8–96.8)
	Negative	25	488				

**FIG 1:**
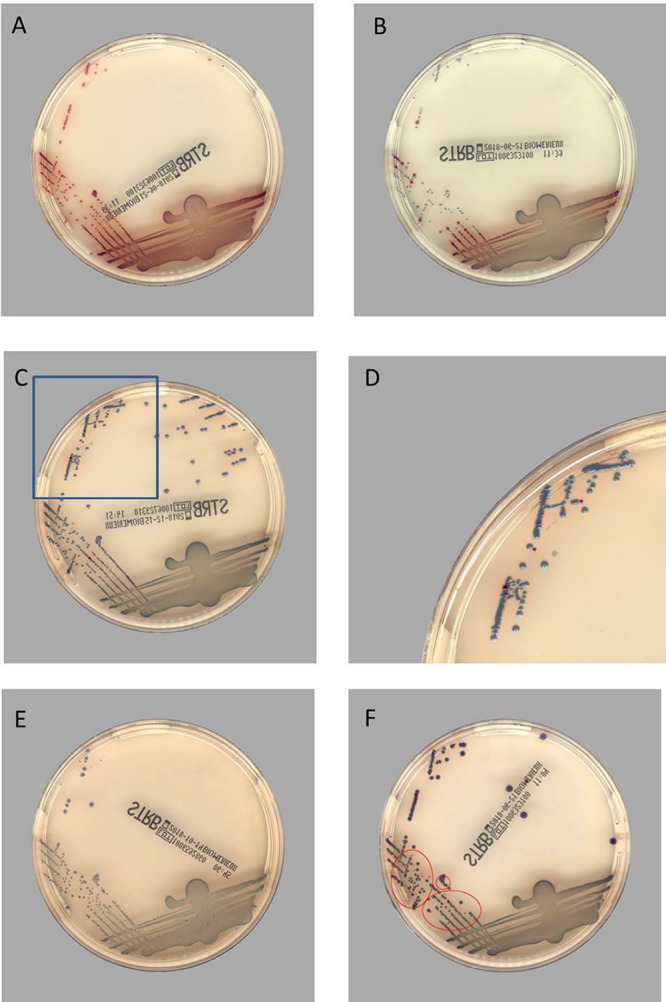
Images of representative cultures on ChromID GBS. (A) PM CDM positive/manual review (MR) positive, GBS high abundance; (B) PM CDM positive/MR positive, GBS low abundance; (C) PM CDM positive/MR negative, GBS true positive; (D) GBS colonies (enlargement of boxed area in panel C); (E) PM CDM negative/MR negative, no GBS; (F) PM CDM positive/MR negative. Isolates were identified as *S. anginosus*.

The PM CDM was then applied to the digital images obtained from the WASPLab instrument, and the results were compared to the results of the manual reading of the ChromID GBS images. Representative images of positive and negative cultures are presented in Fig. 1. At 24 h of incubation, the sensitivity of the PM CDM algorithm compared to the technologist’s reading of the digital image was 99.2%, with one sample being identified as positive by the technologist’s read of ChromID alone but negative by PM CDM. At 48 h, the sample initially not detected at 24 h was detected by the software, resulting in a sensitivity of 100% (Table 2). The specificities of the PM CDM algorithm compared to the technologist’s reading of the digital image were 73.5% and 67.3% at 24 and 48 h, respectively (Table 2). The organisms flagged by the PM CDM as presumptive GBS were consistent with those flagged by technologists for further workup, e.g., *Streptococcus* species and *Enterococcus* species (Fig. 1E and F).

**TABLE 2:** Performance of the ChromID GBS culture compared to the ChromID with PM CDM for detection of presumptive GBS colonies at 24 and 48 h

Incubation time (h)	ChromID result	No. of samples with ChromID + PM result		% (95% CI)			
		Positive	Negative	Sensitivity	Specificity	PPV	NPV
24	Positive	127	1	99.20 (95.1–100)	73.5 (69.6–77.1)	46.1 (40.1–52.3)	99.8 (98.4–100)
	Negative	145	403				
48	Positive	163	0	100.0 (97.1–100)	67.3 (63.0–71.4)	50 (44.4–55.5)	100 (98.6–100)
	Negative	177	336				

A total of 163 samples were positive by both the technologist’s reading and the imaging algorithm. Of these, 139 were true-positive GBS samples. Of the 177 samples that were negative by the technologist’s reading and positive by the imaging algorithm, an additional 8 true-positive GBS were detected (Table 2). Representative images are shown in Fig. 1C and D. Thus, using PM CDM resulted in the detection of 95.5% of the true-positive GBS samples.

Finally, we evaluated each of the methods, ChromID GBS, ChromID GBS plus PM CDM analysis, and GBS NAAT alone. We observed an overall prevalence of GBS of 22.8% (154/676). When all of the methods were compared for performance and adjudication of the discordant results, the sensitivities of the technologist’s reading, the imaging algorithm, and NAAT were 90.3%, 95.5%, and 96.8%, respectively. The specificities of the technologist’s reading, the imaging algorithm, and NAAT were 95.4%, 63.0%, and 97.7%, respectively (Table 3).

**TABLE 3:** Overall performance of ChromID GBS, ChromID GBS with PM CDM at 48 h, and GBS NAAT compared to the reference composite for positivity^*a*^

Test	No. of samples				% (95% CI)			
	TP	FP	FN	TN	Sensitivity	Specificity	PPV	NPV
ChromID	139	24	15	498	90.3 (84.2–94.3)	95.4 (93.1–97.0)	85.3 (78.7–90.2)	97.1 (95.1–98.3)
ChromID + PM	147	193	7	329	95.5 (90.5–98.0)	63.0 (58.7–67.2)	43.2 (37.9–48.7)	97.9 (95.6–99.1)
GBS NAAT	149	12	5	510	96.8 (92.2–98.8)	97.7 (95.9–98.8)	92.6 (87.0–95.9)	99.0 (97.6–99.6)

a TP, true positive; FP, false positive; FN, false negative; TN, true negative.

## DISCUSSION

This study describes the performance of the ChromID GBS medium in conjunction with the PM CDM for the detection of GBS in routine screening cultures compared to the technologist’s reading of a digital image and NAAT. Compared to visual inspection of the digital image by the technologist, the image algorithm was 100% sensitive, with no false-negative results. The algorithm is tuned to detect all presumptive GBS colonies and detected an additional 8 positive cultures that were missed by the technologist’s reading of the culture. Further, we demonstrate that when combined with the PM CDM, ChromID GBS with 48 h of incubation is nearly as sensitive in the detection of presumptive GBS colonies at 48 h (95.5%) as NAAT (96.8%). It should be noted that the package insert of the ChromID GBS agar specifies 24 h of incubation for culture but also states that the sensitivity for detection of GBS may increase at 48 h; however, specificity could be expected to decrease.

Accurate detection of GBS from vaginal-rectal swabs in antenatal screening cultures is imperative for prevention of early-onset GBS disease in neonatal patients. Routine culture on blood agar has a reported sensitivity of 53% to 90% compared to NAAT (8, 10, 15). Some chromogenic media have increased the sensitivity up to 76% (16) compared to NAAT, but this still falls short of the 90.9% to 100% sensitivity attained by using NAAT (9, 10). Use of the PM CDM for the detection of presumptive GBS colonies increases the sensitivity of chromogenic medium alone by using artificial intelligence-aided software to flag colonies that were not detected by the technologist for further examination. In previous studies, algorithmic detection on chromogenic medium demonstrated 100% sensitivity for the detection of presumptive positive cultures for methicillin-resistant Staphylococcus aureus, vancomycin-resistant *Enterococcus*, and group A *Streptococcus* compared to visual inspection (12–14). Here, we compared both the sensitivity and specificity of a newly developed artificial intelligence algorithm-aided culture reading system to those of visual image inspection and to NAAT for the detection of GBS from a new chromogenic agar. Although algorithm-aided culture and NAAT both had false-negative results (7 and 5, respectively), the sensitivity of algorithm-aided culture (95.5%) is similar to that of NAAT (96.8%). The overall sensitivity of algorithm-aided culture was consistent with the reported sensitivities of many of the NAAT methods.

As noted in our results, falsely positive CHROMagar results using PM CDM do occur; however, one must keep in mind that one of the primary purposes of utilizing CHROMagar plus PM CDM is to effectively identify all of the negative GBS cultures with high accuracy. This leaves only the potentially positive cultures for the technologist to review. The negative cultures are grouped by the software, which allows the staff to quickly review up to 40 plates per computer screen and batch-release negative culture results. This is done without the need to review each plate or plate image manually, saving time for the technical staff. Standard laboratory operating protocols would then determine how the potential positive cultures would be resolved. For example, the laboratory’s protocol may call for identification of any potentially positive colony from the CHROMagar by conventional or automated biochemical means, with serological assays, or by MALDI. Studies by Suwantarat et al. (17) and Salimnia et al. (18) have stressed the necessity of confirming GBS organism identification to eliminate false-positive results, especially when chromogenic agars are used (18). As additional chromogenic media are developed which show less deviation in color between strains of GBS, algorithms can continually be modified to achieve greater specificity in addition to the very high sensitivity already being realized.

For the many laboratories that continue to use culture for detection of GBS, a total of 36 to 48 h is required, and a significant amount of manual interaction is necessary for completion. For laboratories that use NAAT to detect GBS, the sensitivity and rapid turnaround time are used to justify the increased cost of the molecular methods. However, routine GBS screening is recommended to be performed within 5 weeks of birth (4), and therefore, when routine screening is performed, the rapid turnaround time offered by NAAT is not required in a majority of cases. In addition, the use of laboratory automation has demonstrated decreased hands-on time for many routine culture types (19, 20).

The cost associated with NAAT for GBS detection may be significant but has been justified for use in some laboratories by the improved sensitivity and workflow of NAAT compared to culture. Although NAAT often has less hands-on-time, reagents are often more expensive and the associated quality control is performed more frequently (i.e., daily) than for chromogenic culture. The cost for a GBS NAAT would likely range from $20 to $30 (all cost amounts are in U.S. dollars) per test, and two levels of quality control would be performed daily for a qualitative test. In a laboratory performing 25 tests per day, Monday through Friday (approximately 250 days/year), with 2 levels of quality control per day, the cost of GBS NAAT testing would be $125,000 to $187,500/year. In contrast, the cost of chromogenic agar is $3 to $5 per plate, and quality control is performed weekly for chromogenic culture media. In the same laboratory, the cost of chromogenic culture for GBS would be $18,750 to $31,250. Laboratory automation with imaging analysis software that does not sacrifice sensitivity may provide a cost-effective alternative to rapid NAAT testing when rapid turnaround time is not required. It is recognized that NAAT may be a reasonable cost-effective alternative for laboratories that have not yet purchased this instrumentation or incorporated imaging automation. Certainly, a significant initial investment in this technology would be necessary in order to achieve the efficiencies associated with PM.

The use of artificial intelligence for colony recognition and presumptive identification in the clinical microbiology laboratory is an emerging field. Colony counting has been available, with significant involvement by the user, for at least 10 years (21). However, in the absence of image analysis integrated into an existing laboratory workflow, there has been limited adoption. With increased automation in the clinical microbiology laboratory, algorithm-aided cultures can exist as part of the routine workflow, with limited interaction by the technologist. As implementation of automation increases along with adoption of algorithm-aided culture reading, there is an opportunity to increase the use of more sensitive, less expensive culture-based methods that have been replaced by NAAT.
